# Role of BAFF in pulmonary autoantibody responses induced by chronic cigarette smoke exposure in mice

**DOI:** 10.14814/phy2.13057

**Published:** 2016-12-30

**Authors:** Mathieu C. Morissette, Yang Gao, Pamela Shen, Danya Thayaparan, Jean‐Christophe Bérubé, Peter D. Paré, Corry‐Anke Brandsma, Ke Hao, Yohan Bossé, Rachel Ettinger, Ronald Herbst, Alison A. Humbles, Roland Kolbeck, Nanshan Zhong, Rongchang Chen, Martin R. Stämpfli

**Affiliations:** ^1^ Department of Pathology and Molecular MedicineMcMaster Immunology Research CentreMcMaster UniversityHamiltonOntarioCanada; ^2^ State Key Laboratory of Respiratory Disease, and Guangzhou Institute of Respiratory DiseaseFirst Affiliated Hospital of Guangzhou Medical UniversityGuangzhouChina; ^3^ Medical Sciences Graduate ProgramMcMaster UniversityHamiltonOntarioCanada; ^4^ Centre de Recherche de l'Institut universitaire de cardiologie et de pneumologie de QuébecQuébecCanada; ^5^ Department of Medicine & Center for Heart Lung InnovationUniversity of British ColumbiaSt. Paul's HospitalVancouverCanada; ^6^ Groningen Research Institute for Asthma and COPD (GRIAC)University of GroningenUniversity Medical Center GroningenGroningenThe Netherlands; ^7^ Department of Pathology & Medical BiologyUniversity of GroningenUniversity Medical Center GroningenGroningenThe Netherlands; ^8^ Department of Genetics and Genomic SciencesIcahn School of Medicine at Mount SinaiNew York CityNew York; ^9^ Department of Respiratory MedicineShanghai Tenth People's HospitalTongji UniversityShanghaiChina; ^10^ Department of Molecular MedicineLaval UniversityQuébecCanada; ^11^ MedImmune LLCGaithersburgMaryland; ^12^ Department of MedicineFirestone Institute of Respiratory Health at St. Joseph's HealthcareMcMaster UniversityHamiltonOntarioCanada; ^13^ Present address: Faculty of MedicineUniversité Laval, Affiliated to CRIUCPQQuébec CityQuébecCanada; ^14^ Present address: Department of Pulmonary and Critical CareBeijing Anzhen HospitalCapital Medical UniversityBeijingChina

**Keywords:** Animal model, autoantibodies, BAFF, cigarette smoke, COPD

## Abstract

Emerging evidence suggests that autoimmune processes are implicated in the pathogenesis of chronic obstructive pulmonary disease (COPD). In this study, we assessed the expression of B‐cell activating factor (BAFF) in smokers, and investigated the functional importance of BAFF in the induction and maintenance of cigarette smoke‐induced pulmonary antinuclear antibodies (ANA) and tertiary lymphoid tissues (TLTs) using a preclinical mouse model. We observed that BAFF levels were elevated in smokers and mice exposed to cigarette smoke. In mice, BAFF expression was rapidly induced in the lungs following 4 days of cigarette smoke exposure and remained elevated following 8 and 24 weeks of exposure. Alveolar macrophages were the major source of BAFF. Blockade of BAFF using a BAFF receptor‐Fc (BAFFR‐Fc) construct prevented pulmonary ANA and TLT formation when delivered concurrent with cigarette smoke exposure. Under these conditions, no impact on lung inflammation was observed. However, administration of BAFFR‐Fc following smoking cessation markedly reduced the number of TLTs and ANA levels and, of note, reduced pulmonary neutrophilia. Altogether, this study shows for the first time a central role of BAFF in the induction and maintenance of cigarette smoke‐induced pulmonary ANA and suggests that BAFF blockade following smoking cessation could have beneficial effects on persistent inflammatory processes.

In this study, we assessed the expression of B‐cell activating factor (BAFF) in smokers, and investigated the functional importance of BAFF in the induction and maintenance of cigarette smoke‐induced pulmonary antinuclear antibodies (ANA) and tertiary lymphoid tissues (TLTs) using a preclinical mouse model. Data presented show that BAFF plays a central role in the induction and maintenance of cigarette smoke‐induced pulmonary ANA and suggest a therapeutic potential for BAFF blockade in limiting autoimmune processes associated with smoking.

## Introduction

Cigarette smoking is the main cause of chronic obstructive pulmonary disease (COPD), a degenerative lung disease with significant impact on pulmonary and cardiovascular health resulting in low quality of life and mortality (Mannino 2002; Hogg 2004; Reardon et al. 2006; Curtis et al. 2007). It is widely accepted that chronic inflammation contributes to the pathogenesis of COPD. Most research, to date, has focused on the innate immune system and the contribution of mediators released by inflammatory cells, such as macrophages and neutrophils, to airflow obstruction and alveolar destruction (Abboud and Vimalanathan 2008). Recently, there is emerging interest in the role of the adaptive immune system in the pathogenesis of COPD. Autoimmune features, such as the presence of systemic autoantibodies, as well as clonal expansion of lung CD4 and CD8 T cells, have been observed in COPD patients and preclinical models of cigarette smoke‐induced inflammation (Lee et al. 2007; Motz et al. 2008; Brandsma et al. 2010; Morissette et al. 2014). We recently reported the presence of broad‐spectrum autoantibodies recognizing antinuclear antigens in the lungs of mice exposed to cigarette smoke; a phenomenon linked with the presence of tertiary lymphoid tissues (TLTs) (Morissette et al. 2014).

B‐cell activating factor (BAFF) encoded by the gene *tnfsf13b* is a member of the tumor necrosis factor ligand superfamily and is instrumental to B‐cell homeostasis and activation (Mackay and Schneider 2009). It binds to three receptors: TACI (*tnfrsf13b*), BCMA (*tnfrsf17*), and BAFF‐R (*tnfrsf13c*), which are mainly expressed by B cells at different developmental stages (Mackay and Schneider 2009). High circulating levels of BAFF have been linked to autoimmune diseases such as systemic lupus erythematosus (SLE), where it is suggested to cause B‐cell hyperactivity and facilitate antinuclear autoantibody (ANA) production (Liu and Davidson 2011). Polverino et al. (2010, 2015) previously showed that BAFF was highly expressed in pulmonary macrophages and B cells of COPD patients compared to healthy controls. Similarly, Seys et al. (2015) reported increased expression of BAFF in the lungs of COPD patients and cigarette smoke‐exposed mice, and that blocking BAFF decreased lung inflammation and tissue destruction in cigarette smoke‐exposed mice.

The main objective of this study was to investigate the role of BAFF in the cigarette smoke‐induced formation and persistence of pulmonary TLTs, and the associated increase in ANAs, in a preclinical model of cigarette smoke exposure. We report that levels of BAFF were increased following short‐term smoke exposure, and remained elevated following chronic exposure to cigarette smoke. BAFF blockade attenuated pulmonary TLT and ANA formation when administered during smoke exposure as well as during smoking cessation. Our study shows a critical role of BAFF in smoking‐induced formation of TLTs and ANA and suggests a therapeutic potential for BAFF blockade in limiting autoimmune processes associated with smoking.

## Methods

### Human samples and analysis of BAFF expression

Messenger RNA levels of *tnfsf13b* (BAFF) were investigated using array analysis in the LAVAL gene expression cohort (Bosse et al. 2012). BAFF protein levels were assessed in the bronchoalveolar lavage fluid (BALF) of cancer‐free nonsmokers and active smokers (Nonsmokers group: 5 males/5 females; aged 49 ± 10 years old; FEV1 104 ± 15% predicted; FVC 108 ± 11% predicted – Active smokers group: 3 males/1 female; aged 60 ± 2 years old; FEV1 99 ± 6% predicted; FVC 114 ± 7% predicted (data are shown as mean ± SD)). Samples were obtained from the Tissue Bank of the Respiratory Research Network of the “Fonds de Recherche du Québec en Santé” (FRQS) at the “Institut universitaire de Cardiologie et de Pneumologie de Québec” (IUCPQ, Laval University, Quebec City, Canada). BALF samples were concentrated using 3K 4 mL Amicon inserts (EMD Millipore, Billerica, MA), and BAFF levels were measured by sandwich ELISA according to the manufacturer's instructions (R&D Systems, Minneapolis, MN). BAFF levels for each sample were normalized to total BALF protein levels as measured with the DC protein assay (Bio‐Rad, Mississauga, ON, CA).

### Animals, cigarette smoke exposure, and BAFF blockade

Six‐ to eight–week‐old female BALB/c mice (Charles River, Montreal, Quebec, Canada) were exposed to cigarette smoke using a whole body exposure system (SIU48; Promech Lab Inc., Vintrie, Sweden) as described previously (Botelho et al. 2010; Morissette et al. 2014). Briefly, mice were exposed twice daily to twelve 3R4F reference cigarettes (Center for Tobacco Reference Products, University of Kentucky, KY), with filters removed, for 5 days per week (Monday–Friday). Control mice were exposed to room air. BAFF blockade was performed by injecting mice intraperitoneally with B‐cell activating factor receptor‐Fc (BAFFR‐Fc) (provided by MedImmune LLC, Gaithersburg, MD) or isotype control (500 *μ*g in PBS) every Monday and Thursday prior to the first cigarette smoke exposure. Timing and duration of BAFFR‐Fc administration are described in detail in the results section and figure legends. Mice were housed in a 12‐h light–dark cycle with food and water ad libitum. All animal protocols were approved by the McMaster University Animal Research Ethics Board (AREB).

### Sample collection and processing

#### Lungs

Mice were anesthetized with isoflurane prior to being killed via exsanguination. Following removal of the lungs from the chest cavity, the left lung (multilobe) was tied off and the right lung (single lobe) was lavaged twice with 250 *μ*L followed by 200 *μ*L of ice‐cold PBS to collect the bronchoalveolar lavage (BAL). Lobes of the left lung were then dissected and snap‐frozen prior to inflation of the right lung with 10% formalin at a constant pressure of 30 cm H_2_O. BAL cell counts were determined using a hemocytometer prior to centrifugation at 500 g for 10 min. Supernatants (BALF) were kept at −80°C and the cell pellet was used to prepare cytospins to assess cell differentials (at least 300 cells counted per cytospin). Formalin‐inflated lungs were washed in ethanol and embedded in paraffin blocks for histological analysis.

#### Pulmonary macrophage isolation and cell culture

Mice were anesthetized with isoflurane prior to being killed via exsanguination. Whole lungs were lavaged five times with 1 mL of sterile ice‐cold PBS. Cells were counted and resuspended in culture media (RPMI 1640, 10% FBS, 2 mmol/L l‐glutamine, 1% penicillin/streptomycin) at a concentration of 5 × 10^5^ macrophages per mL. We previously reported that >90% of cells in the BAL of smoke‐exposed mice are macrophages (Gaschler et al. 2008). In a standard protocol that yields alveolar macrophages in excess of 95% purity (Holt 1979), 5 × 10^4^ macrophages per well were added to a 96‐well plate and incubated at 37°C (5% CO_2_) for 2 h to adhere. Wells were washed with cold PBS three times to remove nonadherent cells, including granulocytes and lymphocytes, and then macrophages were lysed using RIPA lysis buffer. Lysates were collected and stored at −80°C.

### Lung and spleen B‐cell isolation and IgM secretion assay

B cells were isolated from the lungs and spleens of 6–8 week‐old BALB/c mice (Charles River). Lungs were minced in a digestion buffer (HBSS containing 0.75 mg/mL of collagenase A) incubated at 37°C for 1 h. Spleens were crushed between the frosted ends of two glass slides in ice‐cold PBS. Lung and spleen single‐cell suspensions were generated using 40 *μ*m cell strainers. B cells were isolated using EasySep™ Mouse B Cell Isolation Kit (STEMCELL Technologies Inc., Vancouver, British Columbia, Canada). B‐cell purities of 45% and 95% for lung and spleen (CD45^pos^/CD19^pos^/CD3^neg^), respectively, were achieved (data not shown). 10^5^ cells/well were cultured in 96‐well plates in 100 *μ*L RPMI, supplemented with 10% FBS, 2 mmol/L l‐glutamine, 1% penicillin/streptomycin, 0.1% *β*‐mercaptoethanol. Cells were stimulated with mouse recombinant BAFF (R&D systems) or vehicle (PBS) for 72 h. Culture supernatants were collected and IgM levels assessed by ELISA according to the manufacturer's specifications (eBiosciences, San Diego, CA).

### Assessment of mouse BAFF and antinuclear autoantibodies in BAL fluid

Mouse BAFF (R&D Systems) as well as antinuclear autoantibody (ANA) (Alpha Diagnostics Intl. Inc., San Antonio, TX) levels were assessed in the BAL fluid by ELISA according to the manufacturer's specifications.

### Western blot analysis of BAFF in lung tissue and pulmonary macrophage lysates

Lung lobes were homogenized in RIPA lysis buffer with a Polytron homogenizer (Kinematica, Luzern, Switzerland). Total protein concentrations of pulmonary macrophages and lung lysates were determined with the DC protein assay (Biorad, Mississauga, Ontario, Canada). Lysates were resuspended in loading buffer and 15 *μ*g (macrophage) and 40 *μ*g (lung) of proteins were loaded per well. SDS‐PAGE was performed using 10% acrylamide gels and transferred onto nitrocellulose membranes. Membranes were blocked for 1 h (5% fat‐free milk, 0.5% Tween‐20 in PBS) and incubated overnight with rabbit IgG anti‐mouse BAFF (1:1000; Abcam, Cambridge, United Kingdom) and goat IgG anti‐mouse *β*‐actin (1:4000; Abcam) at 4°C. Anti‐rabbit IgG coupled to IRdye 800 nm and anti‐goat IgG coupled to IRdye 700 nm were used as detection antibodies (1:5000, LI‐COR Bioscience, Lincoln, NE). Staining was visualized with the Odyssey CLx Imager (LI‐COR Bioscience). Band intensity was quantified with the Image Studio Lite software (LI‐COR Bioscience) and reported as BAFF intensity over β‐actin intensity for every sample.

### Immunohistochemistry analysis of BAFF and B‐cell localization, and pulmonary TLT assessment

BAFF and B‐cell (B220) detection by immunohistochemistry (IHC) was performed on formalin‐fixed, paraffin‐embedded mouse lung sections. Briefly, lung sections (3 *μ*m) were deparaffinized in xylene and rehydrated in ethanol:water. Endogenous peroxidases were blocked in 3% H_2_O_2_ in methanol. Citrate buffer antigen retrieval was performed (45 min). Sections were blocked with 1% swine serum in Tris‐buffered saline (TBS) with 0.01% Tween 20. Sections were stained with a rabbit IgG anti‐mouse BAFF (1:50; Abcam) or a rabbit IgG anti‐mouse B220 (1:100, Abcam) at 4°C overnight. Primary antibody was detected with a biotinylated goat anti‐rabbit IgG (1:100; 1 h RT) followed by a 30 min incubation with streptavidin‐Horse Radish Peroxidase (Dako, Glostrup, Denmark). Staining was visualized using 3‐amino‐9‐ethylcarbazole (AEC). Pulmonary TLTs were assessed based on morphology as previously described (Morissette et al. 2014). Briefly, three photos at a 16x magnification were taken for every lung at two different cross‐sections. Whole lung cross‐section area (Lung_area_ [pixel^2^/10^10^]), as well as the number of bronchus‐associated TLT (TLT_number_), were determined using Image J Software (https://imagej.nih.gov/ij/index.html) and identified according to their distinctive morphology. Data are expressed as bronchus‐associated TLTs per lung area (TLT_number_/Lung_area_).

### Analysis of lung and spleen B‐ and T‐cell population

Two lung lobes per mouse and the spleen were processed as described in the Methods section under “Sample collection and processing” and “Lung and spleen B‐cell isolation and IgM secretion assay” to obtain a single‐cell suspension. The lung and spleen single‐cell suspensions were assessed for B cell (CD45^pos^ CD3^neg^ CD19^pos^), CD4 T cell (CD45^pos^ CD3^pos^ CD4^pos^ CD8^neg^), and CD8 T cell (CD45^pos^ CD3^pos^ CD4^neg^ CD8^pos^) populations. Flow cytometry was performed using a BD FACS LSR II (BD Biosciences, Mississauga, Ontario, CA) and the analysis using FlowJo software (Ashland, OR).

### Statistical analysis

Statistical differences were assessed using unpaired Student's *t*‐test (2 groups) or one‐way analysis of variance (ANOVA) (>2 groups) followed by a post hoc comparison using a Bonferroni correction for multiple comparisons. Tests were performed using GraphPad Prism (La Jolla, CA).

## Results

### Cigarette smoke exposure induces BAFF expression in the lungs of humans and mice

**Figure 1:**
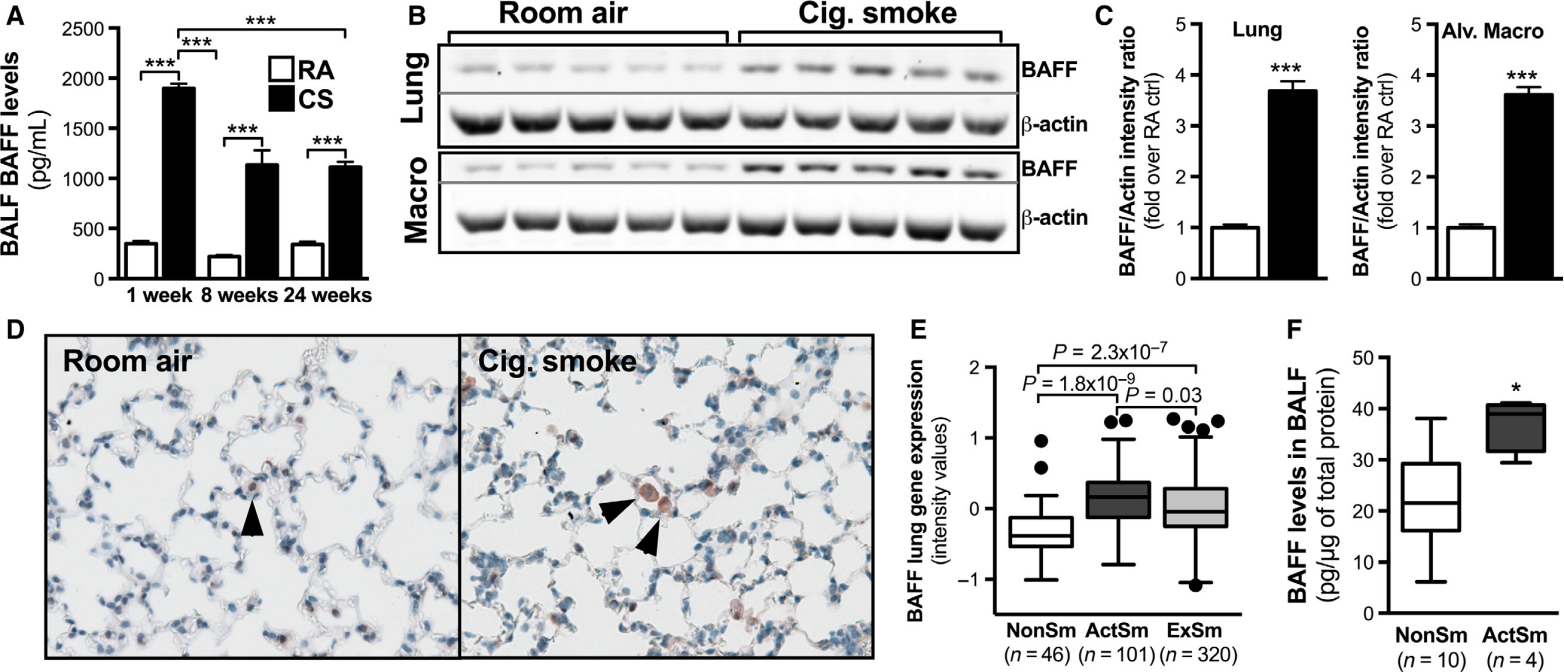
Cigarette smoking leads to an increase in pulmonary B‐cell activating factor (BAFF) levels with pulmonary macrophages as a major source. Female BALB/c mice were exposed to room air (RA) or cigarette smoke (CS or Cig. Smoke) for 4 days, 8 weeks, or 24 weeks. (A) BAFF expression in the bronchoalveolar lavage fluid (BALF). Data show mean ± SEM,* n* = 5/group. (B and C) Representative western blots and quantification of BAFF protein expression in whole lung homogenates and pulmonary macrophage lysates following 4 days of CS exposure. (D) BAFF localization was assessed by immunohistochemistry in the lungs of mice exposed to RA or CS for 4 days. (E) mRNA levels were assessed in resected lung tissues from nonsmokers (NonSm), active smokers (ActSm), and ex‐smokers (ExSm) using gene arrays. (F) BAFF protein levels were measured by ELISA in the BALF of nonsmokers and current smokers. *P < 0.05; ***P < 0.001.

Increased BAFF expression has been reported previously in pulmonary macrophages from smokers and COPD patients (Polverino et al. 2010). We expanded on these observations and investigated BAFF expression in the lungs of mice exposed to cigarette smoke. We observed a marked increase in BAFF levels in the bronchoalveolar lavage (BAL) of mice following 1 week of cigarette smoke exposure (Fig. 1A). Levels remained elevated in cigarette smoke‐exposed animals exposed for 8 and 24 weeks (Fig. 1A). Similar observations were made in whole lung homogenates and pulmonary macrophage lysates (Fig. 1B and C). Immunostaining showed that pulmonary macrophages are a major source of BAFF following cigarette smoke exposure (Fig. 1D). To extend our observations in mice, we analyzed BAFF expression in a gene expression cohort of patients with different smoking status as well as in BAL from current smokers and nonsmokers. In humans, BAFF expression was significantly higher in smokers compared to never‐smokers, and remained elevated following smoking cessation (Fig. 1E). Increased BAFF expression in smokers was confirmed at the protein level in the BAL (Fig. 1F). These data support the observation by Polverino et al. (2010, 2015) and Seys et al. (2015) that BAFF expression is increased in the lungs of smokers and shows that this response is conserved between humans and mice.

### BAFF induces IgM production by splenic and lung B cells ex vivo

**Figure 2:**
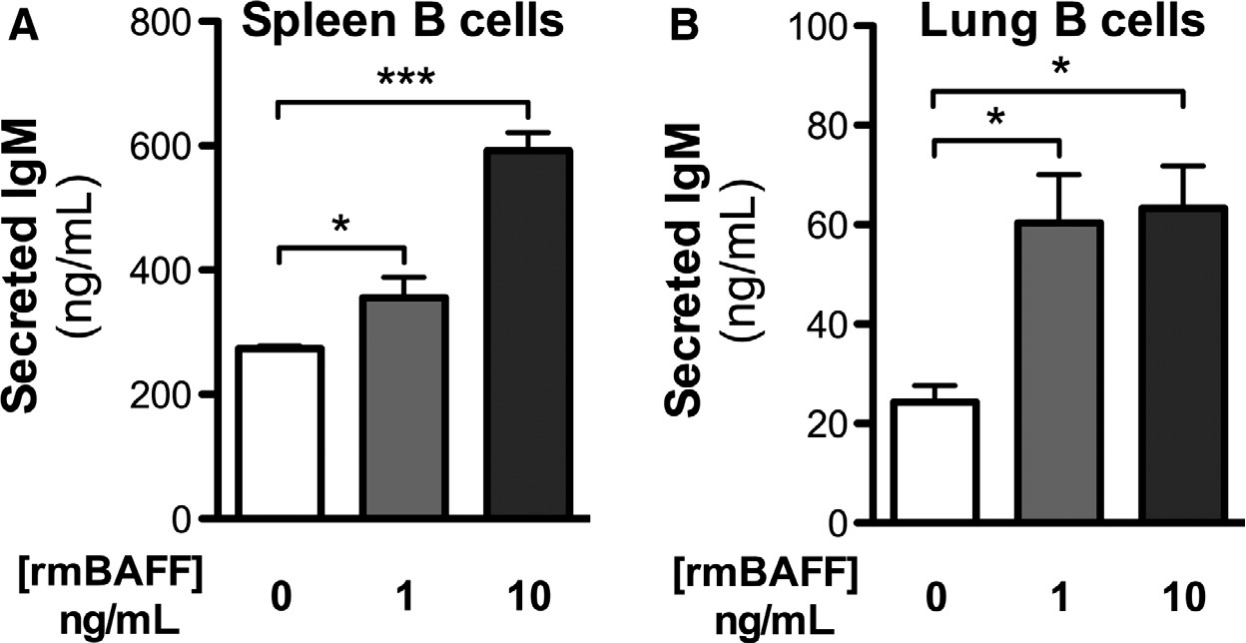
B‐cell activating factor (BAFF) induces antibody production by lung and splenic B cells. B cells were isolated from the (A) spleen and the (B) lungs of female BALB/c mice and stimulated ex vivo for 3 days with recombinant mouse BAFF (rmBAFF). IgM production was assessed in cell supernatants by ELISA. Data show mean ± SEM,* n* = 5/group.; *P < 0.05; ***P < 0.001.

As we observed a marked elevation of BAFF following cigarette smoke exposure, and given the role of BAFF in activating B cells, we sought to investigate whether BAFF was sufficient to induce antibody production by B cells. We found that stimulation of B cells with recombinant BAFF induced a dose‐dependent release of IgM from primary splenic (Fig. 2A) and lung B cells (Fig. 2B). This supports the idea that elevation of BAFF in the lungs contributes to local production of antibodies and, potentially, autoantibodies.

### BAFF neutralization attenuates TLT formation and pulmonary antinuclear autoantibody production in response to chronic cigarette smoke exposure

**Figure 3:**
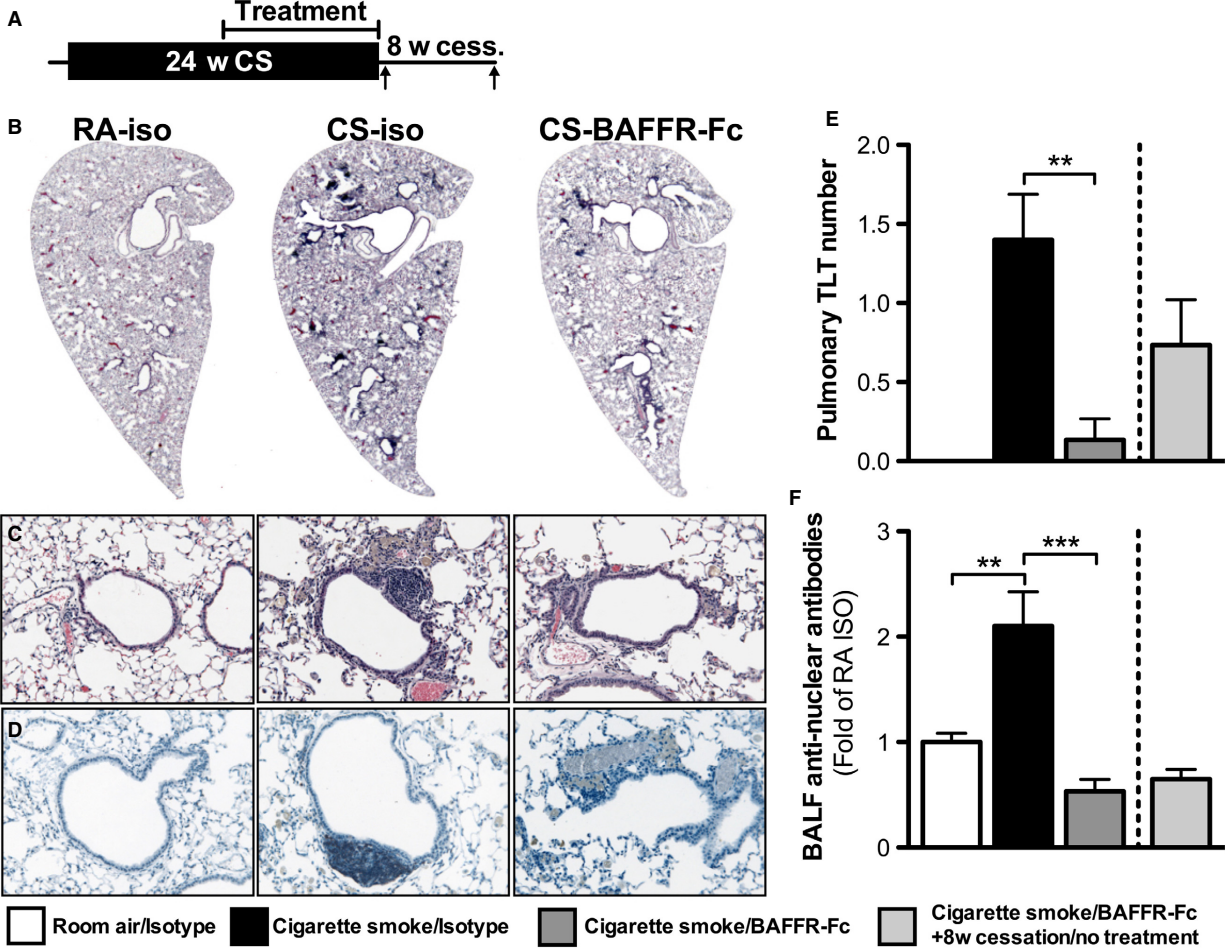
B‐cell activating factor (BAFF) is critical for the formation of tertiary lymphoid tissues and antinuclear antibodies. (A) Female BALB/c mice were exposed to room air (RA) or cigarette smoke (CS) for 24 weeks. BAFFR‐Fc or isotype control was administered intraperitoneally twice a week over the last 12 weeks of CS exposure. Groups of mice were killed 16 h after the last smoke exposure or after 8 weeks of smoking cessation. Representative hematoxylin and eosin (H&E)‐stained lung tissue sections are shown in (B) 16x and (C) 200x magnification. (D) B‐cell (B220+) localization in lung tissue (200x) assessed by immunohistochemistry. (E) Quantification of pulmonary TLT. (F) ANA levels in the bronchoalveolar lavage fluid (BALF). Data represent mean ± SEM,* n* = 5/group.; **P < 0.01; ***P < 0.001.

**Figure 4:**
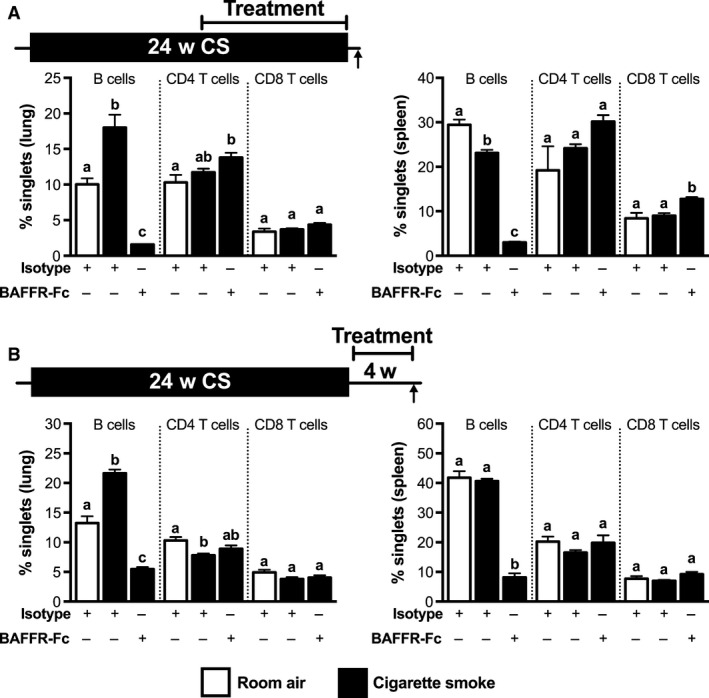
Impact of BAFFR‐Fc treatment on lung and spleen B‐ and T‐cell populations. (A) Female BALB/c mice were exposed to room air (open bars) or cigarette smoke (solid bars) for 24 weeks. BAFFR‐Fc or isotype control was administered intraperitoneally twice a week over the last 12 weeks of cigarette smoke exposure. (B) Female BALB/c mice were exposed to room air (open bars) or cigarette smoke (black bars) for 24 weeks. Following smoking cessation, BAFFR‐Fc or isotype control was administered intraperitoneally for 4 weeks prior to killing. Single‐cell suspensions from the lungs and spleens were analyzed by flow cytometry. Data show B cells, CD4 T cells, and CD8 T cells as percent of the total singlets (mean ± SEM,* n* = 5/group). Bars with different superscripts are significantly different from each other and bars with the same superscript do not differ significantly.

Chronic cigarette smoking induces, in humans and mice, the formation of TLTs and ANA (Hogg et al. 2004; Morissette et al. 2014). To assess the role of BAFF in the formation of TLTs and production of ANA, we exposed BALB/c mice to cigarette smoke or room air for 24 weeks and blocked BAFF using BAFFR‐Fc during the last 12 weeks of exposure (Fig. 3A). A group of mice treated with BAFFR‐Fc was left untreated for another 8 weeks after their last exposure to cigarette smoke as a treatment cessation control (Fig. 3A). As we have previously reported that both TLT and ANA normally persist following smoking cessation in mice that were exposed to cigarette smoke for 24 weeks; we did not include additional cessation control groups (Morissette et al. 2014). Compared to animals treated with the isotype control, mice that received the BAFFR‐Fc had significantly fewer pulmonary TLTs (Fig. 3B, C and D), as shown by H&E, and decreased B cells, as shown by flow cytometry (Fig. 4A). We also observed lower levels of ANAs in the BAL (Fig. 3E). Interestingly, compared to BAFFR‐Fc‐treated mice exposed to cigarette smoke for 24 weeks, there was a slight increase in the number of pulmonary TLTs in BAFFR‐Fc‐treated mice following 8 weeks of smoking and treatment cessation; however, BAL ANA levels remained unchanged. Altogether, these data show that BAFF blockade prevented smoke‐induced pulmonary TLT formation and ANA production when delivered during chronic cigarette smoke exposure.

### BAFF blockade reduces TLT and pulmonary antinuclear autoantibodies following smoking cessation

**Figure 5:**
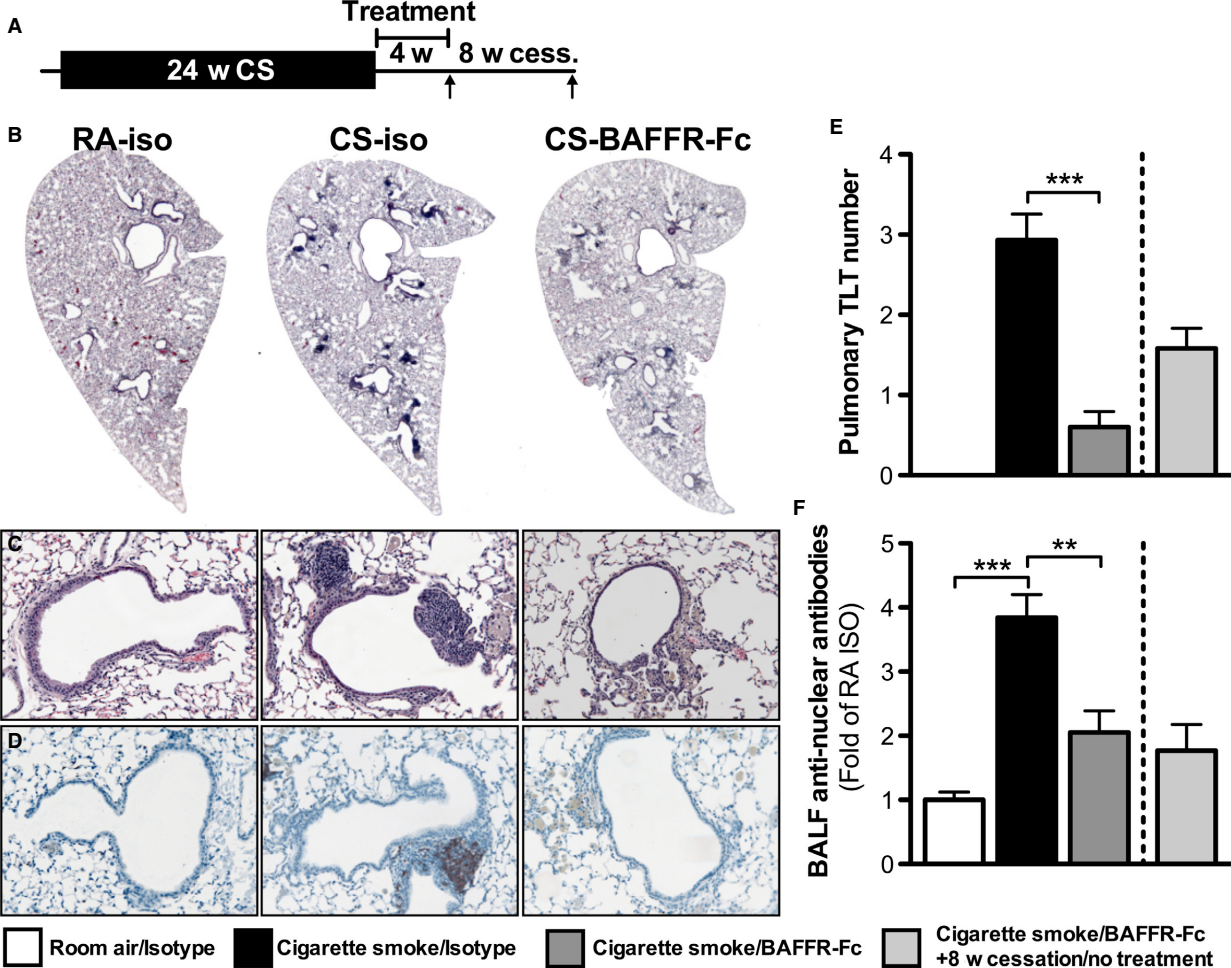
B‐cell activating factor (BAFF) neutralization resolves established tertiary lymphoid tissues and antinuclear antibody responses. (A) Female BALB/c mice were exposed to room air or cigarette smoke for 24 weeks. Following smoking cessation, BAFFR‐Fc or isotype control was administered intraperitoneally twice a week for 4 weeks prior to killing. A group of mice that were smoke‐exposed and received BAFFR‐Fc treatment underwent an additional 8 weeks of no treatment or smoke exposure prior to killing. Representative hematoxylin and eosin (H&E)‐stained lung tissue sections at (B) 16x and (C) 200x magnification are shown. (D) B‐cell (B220) localization in lung tissue (200x). (E) Quantification of pulmonary TLT in lung tissue and (F) ANA levels in the bronchoalveolar lavage fluid (BALF). Data represent mean ± SEM,* n* = 5/group.; **P < 0.01; ***P < 0.001.

Given that BAFF blockade prevented the formation of TLT and ANA induced by chronic cigarette smoke exposure, we next investigated whether BAFF blockade was able to resolve established TLTs and attenuate ANA levels in the lungs following smoking cessation. To this end, we exposed BALB/c mice to cigarette smoke or room air for 24 weeks and administered BAFFR‐Fc for 4 weeks after the last smoke exposure (Fig. 5A). A group of animals were rested for an additional 8 weeks to investigate if the effects of BAFFR‐Fc persisted following treatment cessation (Fig. 5A). Compared to animals treated with the isotype control, mice that received the BAFFR‐Fc had significantly less pulmonary TLTs, as shown in H&E‐stained lung tissues (Fig. 5B, C, and E), and had decreased lung B cells, as assessed by IHC (Fig. 5D) and flow cytometry (Fig. 4B). We also observed decreased levels of ANA in the BALF (Fig. 5E). Moreover, BAFF‐Fc‐treated mice that were left untreated for an additional 8 weeks had only a slight increase in pulmonary TLTs, but no increase in BAL ANA levels. These data suggest that a BAFF blockade resolved established TLTs and attenuated increased ANA levels, and that these effects persisted following treatment cessation.

### BAFF blockade reduces lung neutrophilia following smoking cessation

**Figure 6:**
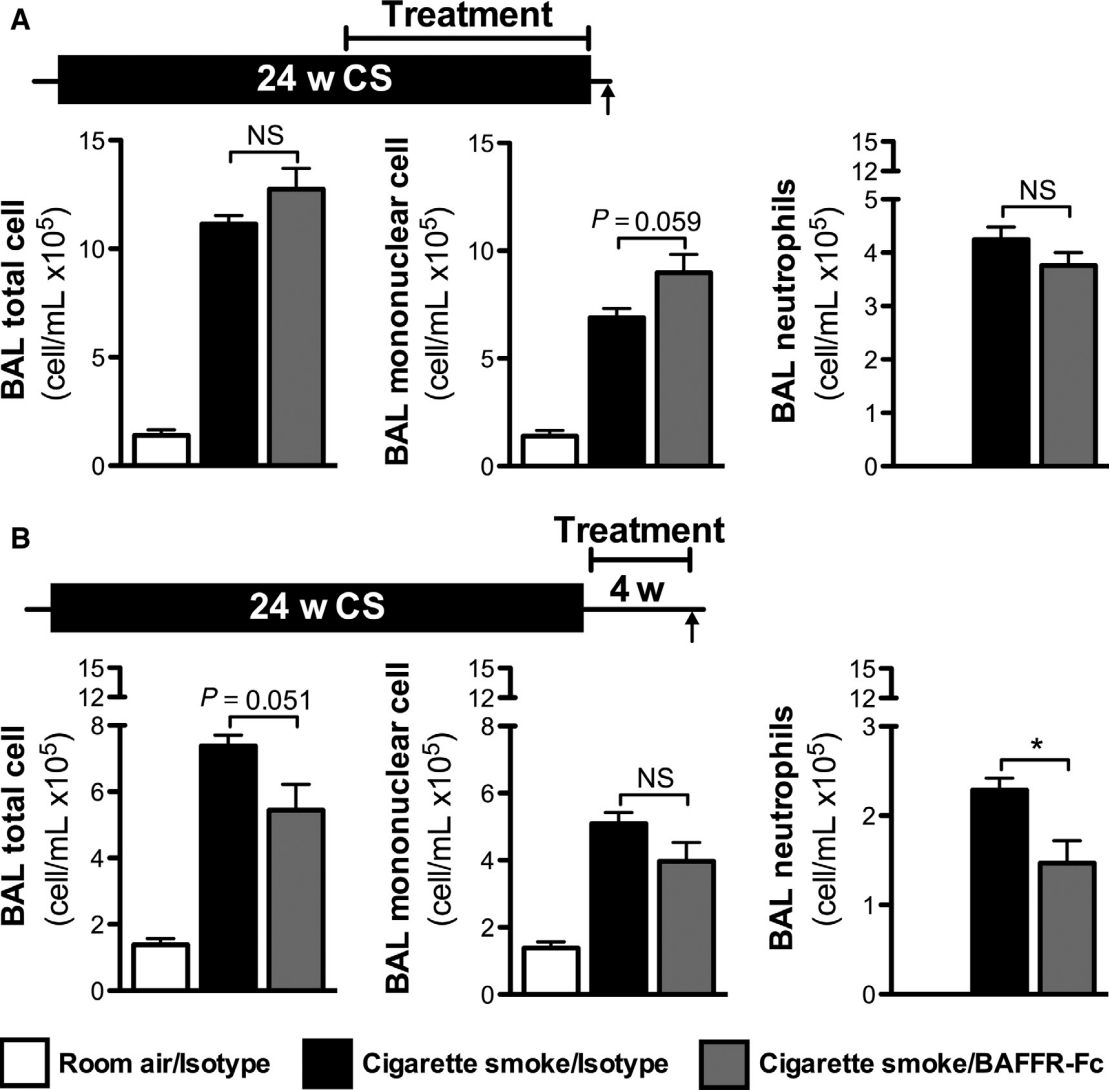
B‐cell activating factor (BAFF) neutralization accelerates resolution of bronchoalveolar lavage (BAL) neutrophilia following smoking cessation. (A) Female BALB/c mice were exposed to room air or cigarette smoke for 24 weeks. BAFFR‐Fc or isotype control was administered intraperitoneally twice a week over the last 12 weeks of cigarette smoke exposure. (B) Female BALB/c mice were exposed to room air or cigarette smoke for 24 weeks. Following smoking cessation, BAFFR‐Fc or isotype controls were administered intraperitoneally for 4 weeks prior to killing. Data show total cell number, mononuclear cells, and neutrophils in the BAL. Data shown as differential cell counts (mean ± SEM,* n* = 5/group). NS, nonsignificant; *P < 0.05.

Cigarette smoke exposure elicits an innate immune inflammatory response that does not fully resolve following smoking cessation (Willemse et al. 2004; Morissette et al. 2014). Therefore, we investigated whether BAFF blockade, and its associated reduction in TLTs and ANAs, impacted inflammatory processes in the lungs. We found that BAFFR‐Fc administration during cigarette smoke exposure had no effect on BAL total cell, mononuclear cell, and neutrophil numbers (Fig. 6A). Of note, BAFFR‐Fc administration following smoking cessation significantly reduced BAL neutrophils, and we observed a trend toward reduced BAL total cell number (*P* = 0.051) (Fig. 6B). These data suggest that BAFF blockade following smoking cessation may promote the resolution of lung inflammatory processes that would otherwise persist.

## Discussion

The adaptive immune system has received recent attention for its potential role in the pathogenesis of COPD. Of particular interest are the presence of autoantibodies and the formation of TLTs in the lungs of COPD patients and mice chronically exposed to cigarette smoke. The objective of this study was to investigate whether BAFF, a potent B‐cell activator, contributed to the production of autoantibodies and the formation of TLT in response to cigarette smoke exposure.

Pulmonary BAFF expression and function was assessed in a well‐characterized mouse model of cigarette smoke exposure (Gaschler et al. 2008; Botelho et al. 2010). We previously reported elevated levels of autoantibodies following 24 weeks of smoke exposure that persisted following smoking cessation, and coincided with the formation of TLT in the lungs (Morissette et al. 2014). We utilized BAFFR‐Fc to block BAFF, using several different experimental protocols, to investigate the function of BAFF in the context of cigarette smoke exposure as well as following smoking cessation. To validate our observations in mice, we analyzed BAFF expression in a human gene expression cohort and BAL samples from nonsmokers, current smokers, and ex‐smokers. The gene expression cohort has previously been described in detail (Bosse et al. 2012).

We observed rapid and sustained increase in soluble BAFF expression in the BAL of cigarette smoke‐exposed mice. These findings suggest that induction of BAFF is part of an early response to cigarette smoke exposure. Similarly to Polverino et al. (2010), we found pulmonary macrophages to be the main source of increased BAFF expression in mice; however, other cells may also produce BAFF in the lungs. Both mouse and human data showed that pulmonary BAFF levels were increased following cigarette smoke exposure alone, suggesting that increased BAFF expression is not solely linked to COPD. While we observed increased BAFF levels within days of cigarette smoke exposure, autoantibodies were only measurable after 8 weeks of exposure. Mechanisms that contribute to this delay in the production of autoantibodies are currently not understood and remain to be elucidated.

BAFF is a potent regulator of B‐cell activation Mackay and Schneider (2009), hence, our observation that BAFF induced antibody production by primary lung B cells suggests that increased expression of BAFF following cigarette smoke exposure may contribute to the production of autoantibodies in vivo. This is in line with the observation that BAFF overexpression induces an autoimmune phenotype in mice characterized by increased autoantibodies against antinuclear targets (Thorn et al. 2010). We are the first to show that BAFF blockade prevented local production of autoantibodies in the lungs of cigarette smoke‐exposed mice. In addition, BAFF blockade attenuated TLT formation, while having no effect on macrophage and neutrophil counts in the BAL. This latter observation is in agreement with Seys et al. (2015) using BAFF‐Fc, as well as Bracke et al. (2013) using CXCL13 blockade. Both these interventions prevented TLT formation, but had no effect on inflammatory read‐outs. In contrast, decreased inflammation was observed in the study by Seys et al. (2015) when BAFFR‐Fc administration was initiated at the same time as smoke exposure. While of interest, this experimental approach is not clinically applicable given that therapeutic interventions will likely be initiated after years of smoking. Taken together, these findings suggest that prevention of autoantibody production and TLT formation may have little impact on the innate inflammatory process induced by cigarette smoke exposure.

Smoking cessation is the most important clinical intervention to prevent the development of smoking‐related diseases, such as COPD. To model the impact of BAFF‐targeted interventions following smoking cessation, we exposed mice to cigarette smoke for 24 weeks and initiated BAFF blockade after smoking cessation. We previously reported increased numbers of TLT and elevated levels of autoantibodies that persist following smoking cessation in mice (Morissette et al. 2014). To the best of our knowledge, this is the first intervention that reduces TLTs and autoantibody levels in the lungs, and accelerates resolution of neutrophilic inflammation following smoking cessation. These data suggest that blocking BAFF signaling may have maximal therapeutic potential when administered in conjunction with smoking cessation. Importantly, pulmonary autoantibody levels remained at baseline even after BAFFR‐Fc administration was discontinued. This suggests that BAFF‐blocking therapies administered for only a short period of time may have the ability to generate long‐lasting beneficial effects, if smoking is not resumed.

In conclusion, this manuscript shows the importance of BAFF in the formation of TLT and the local production of autoantibodies in response to chronic cigarette smoke exposure. Therapeutic blockade of BAFF was effective in preventing, as well as inducing the resolution of TLTs and autoantibodies in the lungs. Further investigations are required to better understand the specific role of TLTs and autoantibodies in COPD to fully justify the development of such targeted interventions.
